# Modern livestock farming under tropical conditions using sensors in grazing systems

**DOI:** 10.1038/s41598-022-06650-5

**Published:** 2022-02-16

**Authors:** Eliéder Prates Romanzini, Rafael Nakamura Watanabe, Natália Vilas Boas Fonseca, Andressa Scholz Berça, Thaís Ribeiro Brito, Priscila Arrigucci Bernardes, Danísio Prado Munari, Ricardo Andrade Reis

**Affiliations:** 1 Department of Animal Science, São Paulo State University (Unesp), Via de Acesso Prof. Paulo Donato Castellane s/n, Jaboticabal, SP 14884-900 Brazil; 2 Department of Engineering and Exact Sciences, São Paulo State University (Unesp), Jaboticabal, SP 14884-900 Brazil; 3 Department of Animal Science and Rural Development, Federal University of Santa Catarina (UFSC), Florianópolis, SC 88045-108 Brazil

**Keywords:** Computational biology and bioinformatics, Animal behaviour

## Abstract

The aim of this study was to evaluate a commercial sensor—a three-axis accelerometer—to predict animal behavior with a variety of conditions in tropical grazing systems. The sensor was positioned on the underjaw of young bulls to detect the animals’ movements. A total of 22 animals were monitored in a grazing system, during both seasons (wet and dry), with different quality and quantity forage allowance. The machine learning (ML) methods used were random forest (RF), convolutional neural net and linear discriminant analysis; the metrics used to determine the best method were accuracy, Kappa coefficient, and a confusion matrix. After predicting animal behavior using the best ML method, a forecast for animal performance was developed using a mechanistic model: multiple linear regression to correlate intermediate average daily gain (iADG) observed versus iADG predicted. The best ML method yielded accuracy of 0.821 and Kappa coefficient of 0.704, was RF. From the forecast for animal performance, the Pearson correlation was 0.795 and the mean square error was 0.062. Hence, the commercial Ovi-bovi sensor, which is a three-axis accelerometer, can act as a powerful tool for predicting animal behavior in beef cattle production developed under a variety tropical grazing condition.

## Introduction

The fourth industrial revolution within agriculture led, around the year 2010, to the concept of precision agriculture or agriculture 4.0^1^. This consisted of introduction of many technologies from the industrial sector that had the aim of improving production and economic results. Within this movement, the last sector to start the fourth revolution was livestock, through creation of the concept of the precision livestock farm^2^. This recently generated concept has been incorporated into different studies aimed at improving sustainable production (animal performance and welfare; and economic, environmental and social results). The main devices, technologies and methods present in these investigations have been computers, sensors, cloud computing, machine learning (ML) and artificial intelligence. Even with all these new perspectives, only 19% of published papers using ML relate to livestock production^3^. Thus, a major gap within which to develop further research exists.

In this regard, some authors highlighted that understanding animals’ feeding behavior within grazing systems could improve management and consequently increase the efficiency of animal production^4^. However, to implement these new technologies, various limitations remain unresolved: these are technological (e.g. internet access, weak signal and absence of an ideal device), scientific (e.g. sensor position, transmission rate, forage management and animal nutrition) and economic (e.g. cost per device and labor efficiency).

In this way, different approaches are being performed to increase prediction to grazing behavior, wherein Barwick et al.^5^ measured sensitivity of grazing behavior attaching sensor on three location (ear, neck, and leg) and achieve values equal to 92%. Other relevant proposal was joining devices, as accelerometers and GPS sensor, which could allow improvement on behavior classifications, as observed by Riaboff et al.^6^, which reported 100% of sensitivity on grazing behavior. Other study showed the promising usage of sensors reporting correlations above 0.68 to different behaviors in beef cattle, using accelerometer and pedometer, under grazing system on organically managed vineyard^7^. Although using only accelerometers, Rayas-Amor et al.^8^ validated this device to grazing and ruminating behaviors on lactating dairy cows kept in a paddock during day and housed at night. Furthermore, different transmission rate was evaluated by Alvarenga et al.^4^, which tested intervals equal to 3, 5 or 10 s of accelerometer transmission to classify sheep ingestive behavior. The authors found accuracy close to 0.79 considering 5 s interval and suggested as the best to predict the main five behaviors studied. Even though previous studies have yielded interesting results, these were based studying only one breed or specific conditions, and some of them concluded that further studies using sensors in grazing systems under different conditions are necessary^9^.Thus, the aim of the present study was to evaluate a commercial sensor—a three-axis accelerometer—to predict animal behavior with a variety of conditions in tropical grazing systems, using different animal genetic groups monitored during both the wet and the dry season, at different beef cattle phases (rearing or finishing).

## Results

The types of animal behavior accounted for different percentages of the total observation record (TOR) from the visual observation *in loco* + video record. These were as follows: 28.9% grazing, 9.8% ruminating, 51.0% lying-standing, 1.4% drinking, 6.0% eating and 2.9% other activities, overall, in the TOR. These values showed that the animal behavior monitored during both phases (rearing and finishing phases) and both seasons (wet and dry seasons) showed unbalanced distribution. The same unbalanced distribution of animal behavior could be seen in each animal group and season (Table 1).

**Table 1:** Types of animal behavior (grazing, ruminating, lying-standing, drinking, eating and other activities) as percentages of the total observation record (TOR), from visual observation *in loco* + video records during both seasons (dry and wet).

Animal behaviour (% TOR)	Animal group^b^			Overall^c^
	Nellore-dry	Nellore-wet	Crossbred-wet	
Grazing	19.9	30.2	56.0	28.9
Ruminating	9.7	0.1	10.8	9.8
Lying-standing^a^	59.0	64.9	25.5	51.0
Drinking	1.5	1.2	1.3	1.4
Eating	7.1	1.7	3.0	6.0
Other activities	2.8	1.9	3.4	2.9

^a^Lying-standing: activities of lying down + standing up. ^b^Animal groups: Nellore-dry: Nellore animals finished during dry season; Nellore-wet: Nellore animals reared during the wet season; Crossbred-wet: crossbred animals reared during the wet season. ^c^Overall: percentage of each type of animal behavior, considering during both phases (rearing and finishing phases) and both seasons (wet and dry seasons).

All the metrics obtained from animal behavior prediction, using different machine learning methods, are demonstrated in Table 2. The best machine learning method was the random forest method, which yielded accuracy of 0.821 and Kappa coefficient of 0.704. These metrics for the other ML methods were, respectively, Accy = 0.626 and 0.596, and κ = 0.336 and 0.283, for CNN and LDA. The confusion matrix metrics for each type of animal behavior showed that, in general, higher metrics were obtained through using the random forest method. The sensitivity measurements from the random forest method were 0.822, 0.675, 0.937, 0.228, 0.411 and 0.417 for the animal behaviors of grazing, ruminating, lying-standing, drinking, eating and other activities, respectively.

**Table 2:** Results from prediction (sensitivity, specificity, precision, accuracy and Kappa coefficient) through machine learning methods (random forest, convolutional neural net and linear discriminant analysis), of the different types of animal behavior observed (grazing, ruminating, lying-standing, drinking, eating and other activities) during both seasons (dry and wet).

Item	Animal behavior						Accy^c^	Kappa^d^
	Grazing	Ruminating	Lying-standing^a^	Drinking	Eating	Other^b^		
**Random forest**							0.821	0.704
Sensitivity	0.822	0.675	0.937	0.228	0.411	0.417		
Specificity	0.932	0.996	0.752	0.999	0.996	0.999		
Precision	0.831	0.954	0.796	0.964	0.863	0.932		
**Convolutional neural net**							0.626	0.336
Sensitivity	0.614	0.072	0.858	0.000	0.045	0.013		
Specificity	0.829	0.995	0.501	1.000	0.997	0.999		
Precision	0.593	0.623	0.643	NA^e^	0.474	0.283		
**Linear discriminant analysis**							0.596	0.283
Sensitivity	0.598	0.000	0.829	0.000	0.017	*		
Specificity	0.798	0.999	0.480	0.999	0.995	**		
Precision	0.549	NA	0.623	NA	0.174	***		

^a^Lying-standing: lying down + standing activities. ^b^Other: Other activities. ^c^Accy: accuracy. ^d^Kappa: Kappa coefficient. ^e^NA: not available. *Sensitivity-other activities = 8.881 × 10^−4^. **Sensitivity-other activities = 9.998 × 10^−1^. ***Precision-other activities = 1.250 × 10^−1^.

Considering only the animal behavior of grazing, the confusion matrix metrics of specificity and precision were 0.932 and 0.831 from the random forest method, respectively; 0.829 and 0.593 from CNN, respectively; and 0.798 and 0.549 from LDA, respectively. For the animal behavior of ruminating, these values were 0.996 and 0.954 from random forest; 0.995 and 0.623 from CNN; and 0.999 and not available (result obtained when the machine learning method did not predict the animal behavior during the analysis) from LDN, respectively for specificity and precision. For the other types of animal behavior (lying-standing, drinking, eating and other activities) the metrics of specificity and precision from each machine learning method were as follows, respectively: 0.752 and 0.796 from RF, 0.501 and 0.643 from CNN, and 0.480 and 0.623 from LDA; 0.999 and 0.964 from RF, 1.000 and not available from CNN, and 0.999 and not available from LDA; 0.996 and 0.863 from RF, 0.997 and 0.474 from CNN, and 0.995 and 0.174 from LDA; and 0.999 and 0.932 from RF, 0.999 and 0.283 from CNN, and 9.998 × 10^–1^ and 1.250 × 10^–1^ from LDA.

**Figure 1:**
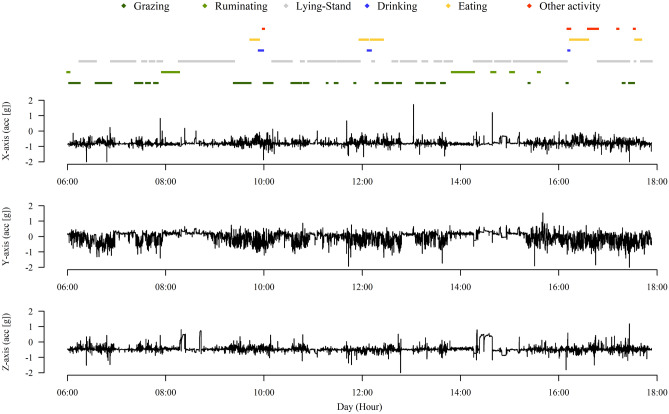
Pattern of records from the X-axis, Y-axis and Z-axis for each type of animal behavior observed (grazing, ruminating, lying-standing^a^, drinking, eating and other activities), from monitoring one animal in this study. (^a^Lying-standing: activities of lying down + standing up).

The imbalance in animal behavior seen in the total observation record (as mentioned above) caused different patterns recorded from each axis (X, Y and Z) for each type of animal behavior (grazing, ruminating, lying-standing, drinking, eating and other activities) from each animal during each observation day (Fig. 1). From these graphs, it was possible to define the times of the day when each type of animal behavior occurred. For example, eating was concentrated at three times (10:00, 12:00 and 16:00 h). Furthermore, the resting position or standing without locomotion reduce the acceleration (ɡ) from all axis (X, Y and Z), in comparison with the other types of animal behavior.

The Pearson correlation from forecasting animal performance between the observed iADG *vs* the predicted iADG was 0.795 and the MSE was 0.062. The summary iADG measurements (maximum, minimum and mean) were respectively 1.552, 0.103 and 1.063 kg for the observed iADG; and 1.602, 0.410 and 1.096 kg for the predicted iADG. More specific results were obtained from each genetic group, with Pearson correlation and MSE of 0.855 and 0.053 for the Nellore groups and 0.637 and 0.084 for the crossbred group, respectively.

**Figure 2:**
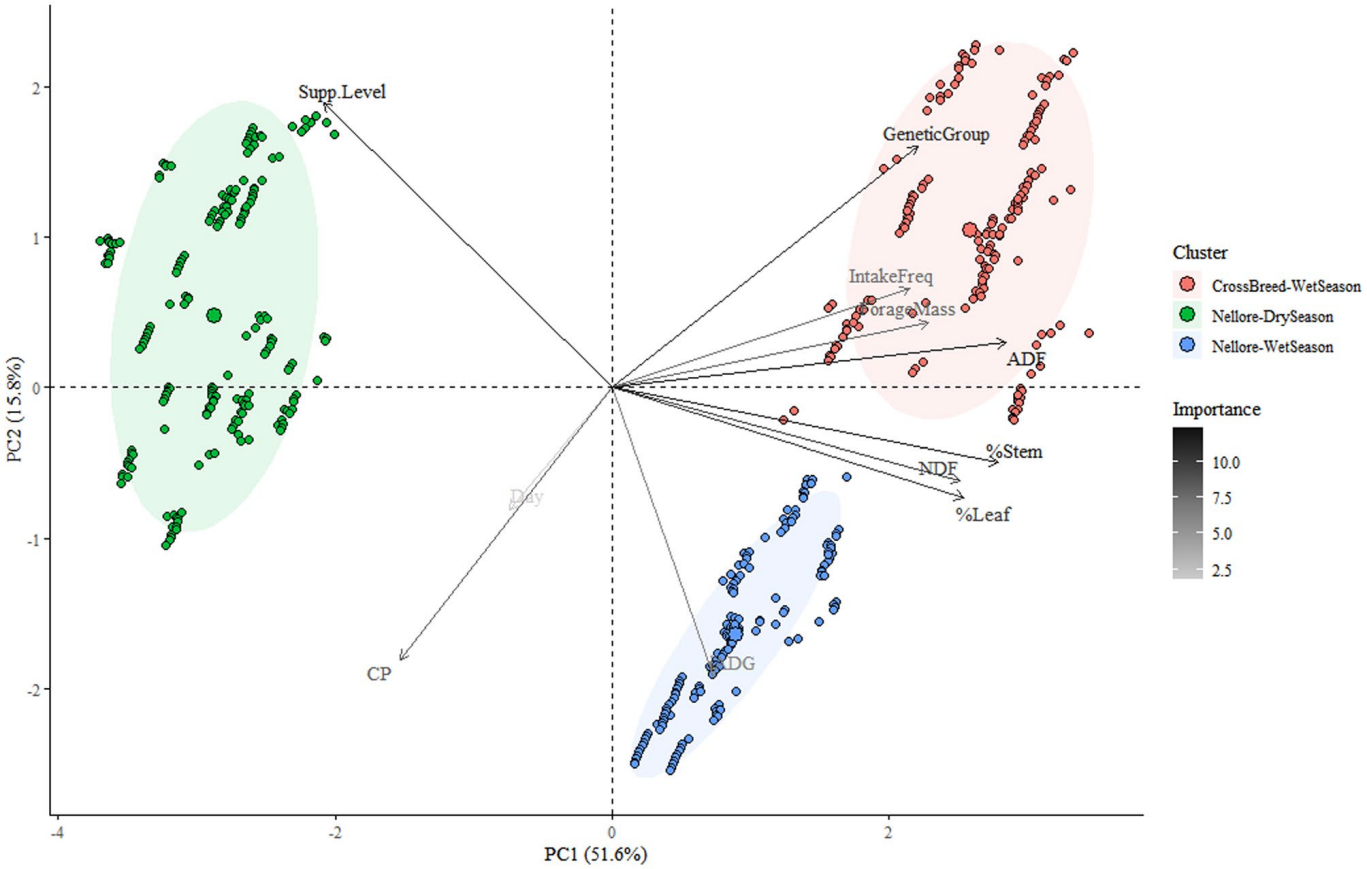
Principal component analysis on the final responses according to their importance for forecasting animal performance and food intake frequency, using a Random Forest method. iADG, intermediate average daily gain; NDF, neutral detergent fiber; ADF, acid detergent fiber; CP, crude protein; PC1, principal component one; PC2, principal component two.

The total data variability in the principal component analysis (Fig. 2) was 67.4%. This was divided into the first principal component (PC1 = 51.6%) and the second (PC2 = 15.8%). The PCA results clearly defined the presence of three clusters, which validated the initial three different animal groups monitored in this study. Figure 2 shows the importance of each variable that was used as an input to the PCA: the lowest importance was observed for the variable of the day and the highest for the supplement level.

## Discussion

The variety of the initial dataset should be highlighted, considering that data collection occurred under different conditions within grazing systems: in the wet and dry seasons, respectively at the animal development phases of rearing and finishing. Thus, this differed from the conditions under which previous studies had been conducted^4,7,10–12^. In those studies, a traditional approach of evaluation of only one specific time, either in a grazing system or not, had been used.

This variety of dataset was an aim for the present study, considering that Brazil has a herd of about 214 million head^13^ that are mainly kept in grazing systems throughout the production chain. In this regard, the frequency of food intake (grazing + eating) predicted from the random forest method was a mean time of 20.35 min h^−1^ (8.14 h day^−1^) spent on this activity. Considering each phase separately, the results were 15.19 min h^−1^ (6.08 h day^−1^) and 20.35 min h^−1^ (8.14 h day^−1^) for the finishing (dry season) and rearing (wet season) phases, respectively.

Recognizing these types of animal behavior is important, given that animals’ performance is directly influenced, in addition to other factors, by their frequency of food intake. Performance can thus be determined with regard to nutritional and non-nutritional factors^14^. Schoenbaum et al.^15^ and Benvenutti et al.^16^ evaluated cattle behavior using other devices in grazing systems. They described differences in the time spent on grazing according to the different conditions, due mainly to forage quality and quantity. The present study also seen this difference, which can be justified due to lower forage mass (5020 kg/ha) available during the dry season, with lower leaf percentage (14.5%), compared to wet season, that presented higher both forage mass (7900 kg/ha) and leaf percentage (30.7%). The knowledge of this variation according to season, lead to adjust the animal management, providing adequate diet supplement level, mainly during dry season. This supplementation aims promotes substitutive effect on forage intake^17^, which replace the main food source, i.e., the animal decreases the forage intake due to higher supplement consumption, which result in lower time spent on grazing, once the chemostatic mechanisms of satiety is achieve instead of physical fill in the rumen^18^. In the other way, during the wet season, the forage quality and quantity allows absence of supplementation (using only mineral mixture) or low levels of supplementation, what lead the animals spent more time grazing, once the main food source is the forage. In this situation, the grazing time will be determined mainly by the physical fulfill of the rumen^18^. These conditions generate in our study, values of 19.9% of grazing behavior on dry season against 30.2% for Nellore and 56% for crossbred on wet season. The metrics from the machine learning method showed that random forest had accuracy of 0.821 for predicting of the studied animal behaviors. This needs to be highlighted because it is determinant for ascertaining the previous time spent. For grazing and eating, respectively, the sensitivity, specificity and precision were 0.822, 0.932 and 0.831; and 0.411, 0.996 and 0.863. If the sensitivity relating to eating behavior is excluded, all the other metrics were above 0.800. Alvarenga et al.^4^ studying accelerometer to classify sheep behavior in only one pasture type reported values above 0.843 for these metrics to grazing and the authors suggested that the model considering transmission interval of each 5 s promote an accurately predicted grazing behavior. Similar value of precision for feeding behavior (0.88) was observed using Original Braunvieh cattle during 15 days of a unique season^7^. Pereira et al.^19^ studying accelerometers to predict grazing behavior of dairy cows reported precision above 0.88 to feeding. Even though the present study used different breed and different pasture conditions in the same model, the results achieved values for grazing behavior close to observed on literature, as well as the general kappa (0.704) can be considered substantial by Landis and Koch^20^ classification, therefore the results can be promising to tropical conditions.

Tedeschi^21^ reported that accuracy and precision are two crucial metrics for evaluating a model, considering the observed versus predicted values. This latter author also stated that accuracy constitutes the ability of the model to predict the right values, while precision is the ability of the model to predict similar values consistently. Thus, considering that the concepts and metrics from the random forest method were the best ones in the present study, good prediction was obtained. Moreover, the bias from visual observation *in loco* + video records, performed by different individuals might, even after training, could be a factor that can lead to obtain lower metrics^8^.

Also, in relation to the metrics from the best machine learning method used for prediction, the lowest sensitivity (0.228) was measured for the animal behavior of drinking. This was probably due to the lower number of occurrences of this type of behavior that were registered through visual observation *in loco* + video records (1.439% of the total raw data). Some authors reported that large datasets had great importance in that they eliminated bias, noise and data imbalance^22,23^. According to the latter authors^23^, a large dataset with large variability, covering as many foreseeable scenarios as possible, should be used for training and testing of machine learning methods. In the present study, changes to natural animal behavior in grazing systems between the two times evaluated were avoided. Thus, the imbalance among all the types of animal behavior evaluated (grazing, ruminating, lying-standing, drinking, eating and other activities) was natural, which can be explained by the fact that the behaviors follow the circadian pattern.

Although the imbalanced behaviors were observed during both season, there were difference on type of behaviors with highest and lowest percentages. The behavior with higher percentage on dry season was lying-standing and with low percentages were drinking and other activities. While on wet season, the percentage of behaviors were different according to animal groups (Nellore or crossbreed), wherein in general, grazing highlight with high percentage and eating with low. The classifiers algorithms tend to easily identify the majority class (behavior with high percentage) and fail to predict the minority class samples (behavior with low percentage) with adequate accuracy^24^. In fact, using our dataset with other purposes, and training the random forest with data from one season to predict on data of the other season, resulted on very low predictive ability. However, the ease of predict the majority class could be observed, wherein training with dry season data to predict on wet season, higher predictive ability for lying-standing behavior (sensitivity 48.5% higher) compared for grazing was measured. The opposite was observed when using to train the dataset from wet season to predict the behaviors on dry season, with the same quantity of data. In this case, higher predictive ability for grazing (sensitivity 2.3% higher) was noted when compared to lying-standing behavior. The behavior with high percentage on training data was less difficult to predict. In both cases, the less frequent behaviors were harmed, with low predict ability. In this way, join the data from different conditions in unique dataset to train the algorithm, allows improve the accuracies in general, as showed in this study.

Animal behaviors were then predicted using the random forest machine learning, which was called “hybridization” by Ellis et al.^22^. This was because the responses from the machine learning method are used as the input to a mechanistic model, i.e. multiple linear regression, from which in this case forecasting of animal performance, measured by the intermediate average daily gain (iADG) is obtained. According to Moretti^25^, even when animals undergo a fasting period, a range of body weight increase from 4 to 12% may occur. Therefore, in the present study, the animals were weighed without having undergone a previous fasting period, and this was also done in another research. Even using the iADG and with low number of animals to forecast animal performance, which is not recommended, this study tried to suggest different approach that predicted behaviors by accelerometers can be used. The Pearson correlation obtained through comparison of the observed iADG versus the predicted iADG was 0.795, which was promising. According to Hopkins^26^, who described a scale of magnitudes for statistical effects, the present value would be classified as very large. Separate forecasts of animal performance for the Nellore and crossbred animals using the same scale showed that the value for the Nellore animals was classified as very large, for both seasons (wet and dry) and both developmental phases (rearing and finishing). The value for the crossbred animals was classified as large.

The frequency of food intake is one of the many factors that can influence average daily gain. Since the MLR to predict iADG considered nutritional value of forage, supplement level and day, the animals in the same level of these factors were in the same phase of life and similar physiologic condition. Therefore, the individual behavior of frequency of food intake that each animal developed, both within and between the genetic groups, can explain the differences observed for Pearson correlation on studied breeds. These potentials that exist both between genetic groups and between weather conditions can cause changes to frequency of food intake among animals^27^. Also in this regard, evaluating Holstein Friesian cows that were kept in barns and in a grazing system, research reported that there was a major individual effect on the time spent on grazing and ruminating^28^. A final validation was observed from principal component analysis, consisting of an unsupervised learning method that was performed using all the final results from the hybridization process. This resulted in clustering comprising three clusters, which were precisely the same three animal groups that were initially monitored (Nellore—dry season; Nellore—wet season; and crossbred animals—wet season). This result showed that through using only the predicted values from the machine learning method (random forest) and the mechanistic model (MLR), and the feed nutritional value, the real condition was proven.

The traditional method of predicting dry matter intake presents various difficulties, especially among ruminants^29^. Although the approach of the present study was not to obtain dry matter intake, the observing the results of the present study showed that the frequency of food intake predictions can be performed easily. Here, sensors comprising three-axis accelerometers produced with real-time records that were input to hybridization using a machine learning method (random forest) plus a mechanistic model (MLR). This resulted in a large amount of information and knowledge that can be used by farmers to improve their decision-making regarding the beef cattle production chain, developed under tropical grazing conditions.

Hence, the precision livestock farm has become a reality boosted through Agriculture 4.0, using technologies like sensors, big data and machine learning (artificial intelligence). The commercial Ovi-bovi sensor, which is a three-axis accelerometer, can act as a powerful tool for predicting animal behavior on variety of conditions in tropical grazing systems. The use of this prediction seems promising for forecasting animal performance, although more studies are recommended, aiming to provide information for farmers and researchers that enables higher production with greater efficiency under tropical rangeland.

## Methods

All the procedures used followed the Ethical Principles for Animal Experimentation stated by the National Council for Animal Experiment Control and were approved by the Ethics Committee for Use of Animals (CEUA) of São Paulo State University (Unesp) (under protocol #001081/2019). Furthermore, this study followed all the applicable procedure in accordance with the Animal Research: Reporting of In Vivo Experiments (ARRIVE) guidelines^30^.

### Experimental area and data collection

The field phase of this experiment was developed during both the dry and the wet season, under tropical conditions, at the Forage Crops and Grasslands section of São Paulo State University “Júlio de Mesquita Filho” (Unesp), in São Paulo, Brazil. The typical climate of this region is subtropical humid, with dry winters and wet summers. Pastures were sown with *Urochloa brizantha* (Hochst.) Stapf, commonly known as palisade grass or “Marandu” grass. The total pasture area of Forage Crops and Grasslands section comprised 20 hectares (ha), where only nine paddocks of different areas were used in this study: one paddock of 0.50 ha; five paddocks of 0.65 ha and three paddocks of 0.70 ha.

Three different animal groups were used to obtain all the sensor records: two groups of Nellore breed and one crossbred group (½ Nellore + ½ Angus). One Nellore group was finished during the dry season by feeding with a high level of supplementation: 2.0% BW (ingredients on Supplementary material—Table), composed by 17.4% crude protein (CP), 13.8% neutral detergent fiber (NDF), 5.7% acid detergent fiber (ADF) and 15.9% gross energy (GE). The other groups were reared during the wet season: the Nellore group was fed without supplementation (using only a mineral mixture); and the crossbred group was fed with a supplement at the level of 0.3% BW (ingredients on Supplementary Table S1), formulated using two different energy sources (corn and citrus pulp), wherein the supplement with corn was composed by 10.6% CP, 11.4% NDF, 5.3% indigestible NDF and 85% total digestive nutrients (TDN), while the supplement with citrus pulp was composed by 8.3% CP, 30.2% NDF, 7.5% indigestible NDF and 77% TDN. All the management practices used at both times (rearing and finishing phases) followed moderate intensification criteria with continuous grazing, as described by Cardoso et al.^31^.

Data were gathered at two times: September and October 2019 (dry season); and January to March 2020 (wet season). A total of 22 animals with average age around 15 months were monitored by means of sensors: ten Nellore animals (343 ± 27 kg) during the dry season, handled using 3.0 animal unit per hectare (AU/ha); and five Nellore (310 ± 38 kg), handled using 5.9 AU/ha, and seven crossbred animals (324 ± 37 kg), handled using 4.6 AU/ha, during the wet season. For all the animals, there was an adaptation period of a minimum of seven days before the experiment was started, to accustom them to wearing a halter with a sensor attached. The animals monitored in the experiment during 78 days on the wet season were in the rearing phase, while those that were monitored during 19 days on the dry season were in the finishing phase. During the dry season, the ambient mean temperature, maximum mean temperature, and minimum mean temperature were 26.1 °C, 34.0 °C and 18.5 °C, respectively, and the rainfall was 157 mm distributed over 12 days, producing an average of forage mass (FM) equal to 5020 kg/ha with 1.6 leaf to stem ratio, 16.5% CP, 51.5% NDF and 24.9% ADF. During the wet season, these temperatures were 24.9 °C, 30.5 °C and 19.4 °C, respectively, and the rainfall was 627 mm distributed over 44 days, which produced on average 7900 kg/ha of FM with 1.0 leaf to stem ratio, 15.6% CP, 61.3% NDF and 30.2% ADF.

### Sensors and animal behavior

The sensors used in this study were three-axis accelerometers using a microelectromechanical system (MEMS) (model LIS2DE12; ST Microelectronics), supplied in the form of a commercial device from the Ovi-bovi company. The device weighed 80 g, with dimensions of 105 mm × 60 mm × 22 mm, and was attached to a halter and positioned on the underjaw of the young bulls to detect their movements, similar to what was described by Watanabe et al.^32^. The sensors were used to record movements along the three axes (X [horizontal movements − side to side], Y [longitudinal movements − front to back] and Z [vertical movements − up and down]) throughout the day and night (24 h).

Each record was made in real time over a period of 6 s (approximately 0.167 Hz), which was defined considering study purpose and battery usage following manufacturer. These records were collected using a wireless system (band of 433 MHz) and stored in the company server (Ovi-bovi). The raw data were then accessed and stored for use in the present study, after conversion from their decimal format to ɡ units (ɡ = 9.81 m s^−2^).

Information on real animal behavior was obtained from two sources: direct visual observations *in loco*^33^ during 2 days on dry season and 4 days during wet season totalizing 72 h and recordings using a video camera (Xtrax model Xtrax Smart2) during 3 days on wet season totalizing 8 h. For both of these sources, records were made every 10 min^34^. A total of 80 h of observations were made over a 12-h period per day (06:00 to 18:00 h) in both seasons (dry and wet), as described by Barbero et al.^35^. A total of six types of animal behavior were observed: grazing, ruminating, lying down + standing up (lying-standing), drinking, eating (ingesting dietary supplement) and other activity (Table 3). In general, one training person was responsible for observing a maximum of three animals kept in different paddocks, inside the experimental area described previously.

**Table 3:** Classification for registering different types of animal behaviour.

Animal behavior	Characterization
Grazing^a^	Animals searching for food while walking short distances with their head down, without picking food up with their mouth; standing still with their head down while apprehending food with their mouth; and chewing either with their head down or their head up, while stationary
Ruminating	Animals chewing and swallowing a ruminal bolus
Lying-standing^b^	Animals lying down in any resting position and animals standing up on all four legs, without locomotion
Drinking	Animals putting their mouth in a water drinker and swallowing
Eating	Animals located in the feeding supplement zone, ingesting dietary supplement
Other activities	Animals doing activities other than those described above

^a^Supplementary Video S2. ^b^Lying-standing: activities of lying down and standing up (Alvarenga et al.^4^, Poulopoulou et al.^7^).

### Machine learning methods

The initial dataset used in each machine learning procedure was the same. It was composed of variables from the sensor, the animal’s identification, time data and calculated variables. The sensor variables comprised the axis records (X, Y and Z); the animal identification variables were the SensorID and genetic group and the time data variables were the day, month, hour, minute and second. The calculated variables (signal magnitude area (SMA), signal vector magnitude (SVM), movement variation, energy, entropy, pitch, roll and inclination) were obtained through the methods described by Alvarenga et al.^4^, using the axis records from the sensor (Table 4).

**Table 4:** Calculation of variables from the sensor axis records.

Variable	Equation
SMA^a^	$\left|{X}_{i}\right|+\left|{Y}_{i}\right|+\left|{Z}_{i}\right|$
SVM^b^	$\sqrt{{X}_{i}^{2}+{Y}_{i}^{2}+{Z}_{i}^{2}}$
Movement variation	$\left|{X}_{i+1}-{X}_{i}\right|+\left|{Y}_{i+1}-{Y}_{i}\right|+\left|{Z}_{i+1}-{Z}_{i}\right|$
Energy	${\left({X}_{i}^{2}+{Y}_{i}^{2}+{Z}_{i}^{2}\right)}^{2}$
Entropy	${\left(1+\left({X}_{i}+{Y}_{i}+{Z}_{i}\right)\right)}^{2}\times ln \left(1+{\left({X}_{i}+{Y}_{i}+{Z}_{i}\right)}^{2}\right)$
Pitch (degrees)	$tan^{ - 1} \left( {{{ - X_{i} } \mathord{\left/ {\vphantom {{ - X_{i} } {\left( {\sqrt {Y_{i}^{2} + Z_{i}^{2} } } \right)}}} \right. \kern-\nulldelimiterspace} {\left( {\sqrt {Y_{i}^{2} + Z_{i}^{2} } } \right)}}} \right) \times 180{/}\pi$
Roll (degrees)	$atan2(Y_{i} ,Z_{i} ) \times 180{/}\pi$
Inclination (degrees)	$tan^{ - 1} \left( {{{\left( {\sqrt {X_{i}^{2} + Y_{i}^{2} } } \right)} \mathord{\left/ {\vphantom {{\left( {\sqrt {X_{i}^{2} + Y_{i}^{2} } } \right)} {Z_{i} }}} \right. \kern-\nulldelimiterspace} {Z_{i} }}} \right) \times 180{/}\pi$

^a^SMA, signal magnitude area; ^b^SVM, signal vector magnitude (Alvarenga et al.^4^).

After calculation of all these variables, a quality control process was performed to improve the dataset information that would be used to evaluate all the machine learning methods. The quality control basically considered a maximum interval between successive raw data records. Given that the sensors were configured to record information relating to successive six-second periods, this control considered an interval of 60 s as a maximum. When the successive raw data records were higher than 60 s, a new sequence was started for successive raw data records, for all the variables previously mentioned.

For all the machine learning methods evaluated, the dataset was divided into two subsets (training and validation). This process was developed to avoid overfitting. From the total dataset, 70% was considered to be a training dataset and 30% was used to validate the ML method that had previously been trained using the training set. In this way, the ML methods evaluated in the present study were random forest (RF), convolutional neural net (CNN) and linear discriminant analysis (LDA). The predictive ability of each machine learning method was measured using a confusion matrix (sensitivity, specificity and precision [Eqs. 1–3]), accuracy (Accy [Eq. 4]) and Kappa coefficient, obtained through using the “caret” package from R Core Team^36^.

1 $$Sensitivity = True \;Positive{/}(True\;Positive + False\;Negative)$$

2 $$Specificity = True\;Negative{/}(True\;Negative + False\;Positive)$$

3 $$Precision = True\;Positive{/}(True\;Positive + False\;Positive)$$

4 $$Accy = \frac{(True\;Positive + True\;Negative)}{{(True\;Positive + True\;Negative + False\;Positive + False\;Negative)}}$$

where True Positive was the number of instances in which the animal behavior of interest was correctly classified after validation; False Negative was the number of instances in which the animal behavior of interest was observed visually but was classified incorrectly as some other animal behavior; False Positive was the number of instances in which the animal behavior of interest was incorrectly classified but not observed; and True Negative was the number of instances in which the animal behavior of interest was correctly classified as not being observed.

Kappa coefficients (κ) compare the observed accuracy with the expected accuracy (random chance). Landis and Koch^20^ presented a classification system for kappa values, in which the correlation was defined as poor (κ < 0.000), slight (0.000 < κ < 0.200), fair (0.201 < κ < 0.400), moderate (0.401 < κ < 0.600), substantial (0.601 < κ < 0.800) or almost perfect (0.801 < κ < 1.000).

### Random forest

The random forest method was performed in the R Software^36^ using different packages within the software, like “randomForest”, “dplyr” and “e1071”. All the variables previously mentioned were used in this analysis on machine learning. The model characteristics (*mtry*, *nodesize* and *maxnodes*) were kept as defaults from “randomForest” package, but the *ntree* characteristic was defined as 500. A tuning process was developed but no differences were obtained. Therefore, it was decided to use the default values.

### Convolutional neural net

The convolutional neural net method with one dimension was performed in the R Software^36^ using the “keras” and “Tensorflow” packages. This was done in the form “Keras + Tensorflow”. The model architecture used in this evaluation had three layers with different neuron numbers (24, 12 and 6; respectively for each layer). The activation function at the first two layers was *relu*, whereas at the last layer, *softmax* was used as the activation function. Other important information about the model structure included the *number of epochs, validation_split* and *batch_size*, which were 500, 0.2 and 128, respectively. Lastly, the optimizer used in this model was *adam*.

### Linear discriminant analysis

The linear discriminant analysis, like other machine learning methods, was performed in the R Software^36^ using a package called “MASS”. For this machine learning method, the entire analysis was performed using a default from the package, to predict the different types of animal behavior evaluated in this research.

### Forecasting animal performance

An approach of animal behaviors information obtained by accelerometers different of health and welfare application could be the prediction of animal performance, which can assistant management in farm. Therefore, the present study evaluated a simple forecasting animal performance using this information as an idea to be developed. After the best machine learning method for predicting animal behavior has been defined, a new prediction was developed with the aim of obtaining the intermediate average daily gain (iADG) from each animal. For this forecasting, data from all the 22 animals that had been monitored with sensors were used. The iADG was calculated using the weight measured at the start and at the end of each experimental period (which was defined each 28 days following other research that was developed in conjunction with the present study), when the animals were weighed without fasting, as commonly recommended to obtain the final ADG.

The animal performance forecasting was developed using multiple linear regression (MLR)^37^. In addition to intake frequency, which was previously obtained through machine learning predictions for each animal, the following other variables were used at this time: genetic group, day, supplement level and some information about forage, including crude protein (CP), neutral detergent fiber (NDF), acid detergent fiber (ADF), forage mass and percentages of leaf (%Leaf) and stem (%Stem). All of this information about forage was obtained through other research that was developed in conjunction with the present study, following methods described by Koscheck et al.^38^.

In the same way as in the evaluation of machine learning methods, the dataset for this phase was divided between training and validation datasets, using the same percentages previously mentioned for each dataset. The Pearson correlation (r) and mean square error (MSE) were used to measure the predictive ability of MLR to forecast animal performance. This correlation took into account the observed iADG (real value measured in the field) *vs* the predicted iADG (value obtained from the MLR model). The MSE criterion used the values of the predictive variables associated with future observations and with the magnitude of the estimated variance^39^. The same evaluations were also made taking the genetic groups into account, with the aim of observing the forecasts for animal performance in each of these groups. All of these evaluations were developed using the R Software^36^.

### Multivariable analysis

Principal component analysis (PCA) was performed using all the variables present in the final dataset used for forecasting animal performance. This dataset was composed of intake frequency, iADG, supplement level, genetic group, forage mass, CP, NDF, ADF and %Leaf and %Stem. The PCA was performed using the “factoextra” package of the R Software^36^. From these settings, factor analyses were obtained using PCA, which were calculated using a correlation matrix of the variables^40,41^. Through this, the total data variability from the dataset was measured and, consequently, the presence or absence of clusters was defined.

## Supplementary Information

Supplementary Table S1.

Supplementary Video S2.

Supplementary Information.

## Data Availability

The datasets generated during and/or analyzed during the current study are available upon request to the corresponding author. The files contain since animal behavior until sensor records, as described in “Methods” section. The datasets are in extensions: *.xls, *.xlsx, *.RData.
