# Supplementary material for: Modern livestock farming under tropical conditions using sensors in grazing systems
Source: Sci Rep. 2022 Feb 16;12:2654. doi: 10.1038/s41598-022-06650-5 (PMC8850600; doi:10.1038/s41598-022-06650-5)
Supplement: Supplementary file 1 — Supplementary Table S1. [file 41598_2022_6650_MOESM1_ESM.docx]

**Supplementary Table S1.** Supplement diet composition provided for animals during both season (dry and wet) evaluated in this study.

| Ingredient (%DM^1^) | Animal group (Supplement diet) | | | | |
| --- | --- | --- | --- | --- | --- |
|  | Nellore - Dry | Nellore - Wet | Crossbred -Wet | |  |
|  | Supplement^2^ | Mineral Mixture^3^ | Supplement^4^ (Corn basis) | Supplement^4^ (Citrus pulp basis) |  |
| Corn ground | 87.0 | - | 95.0 | - |  |
| Soybean meal | 3.5 | - | - | - |  |
| Cottonseed meal | 3.9 | - | - | - |  |
| Citrus pulp | - | - | - | 95.0 |  |
| Urea | 1.6 | - | - | - |  |
| Mineral Mixture | 4.0 | 100.0 | 5.0 | 5.0 |  |
| Total | 100.0 | 100.0 | 100.0 | 100.0 |  |

^1^DM: dry matter; ^2^Supplement provided at 2.0% of body weight (BW); ^3^Supplement provided *ad libtum*; ^4^Supplement provided at 0.3% BW
