# Supplementary material for: Modern livestock farming under tropical conditions using sensors in grazing systems
Source: Sci Rep. 2022 Feb 16;12:2654. doi: 10.1038/s41598-022-06650-5 (PMC8850600; doi:10.1038/s41598-022-06650-5)
Supplement: Supplementary file 2 — Supplementary Video S2. [file 41598_2022_6650_MOESM2_ESM.docx]

**Supplementary Video S2.** Crossbreed animal during a grazing behavior at wet season in a tropical rangeland
